# Atrial Fibrillation is Associated with Femoropopliteal Totally Occlusive In-Stent Restenosis: A Single-Center, Retrospective, Observational Study

**DOI:** 10.1155/2021/8852466

**Published:** 2021-02-03

**Authors:** Yohsuke Honda, Keisuke Hirano, Masahiro Yamawaki, Motoharu Araki, Norihiro Kobayashi, Yasunari Sakamoto, Shinsuke Mori, Masakazu Tsutsumi, Kenji Makino, Shigemitsu Shirai, Masafumi Mizusawa, Takahiro Nakano, Yoshiaki Ito

**Affiliations:** Department of Cardiology, Saiseikai Yokohama-City Eastern Hospital, Yokohama, Japan

## Abstract

**Introduction:**

The proportion of patients with comorbid atrial fibrillation (AF) and peripheral artery disease (PAD) has increased in this era. This study aimed to assess the relationship between AF and totally occlusive in-stent restenosis (ISR) in femoropopliteal (FP) lesions.

**Methods:**

In this study, 363 patients (461 stents) who underwent endovascular therapy with de novo stent implantation in our hospital between April 2007 and December 2016 were retrospectively evaluated. The patients were divided into two groups according to the AF status (AF group, 61 patients; sinus group, 302 patients). The primary endpoint was the incidence of totally occlusive ISR within 3 years. The secondary endpoint was the incidence of acute limb ischemia (ALI) due to FP stent occlusion.

**Results:**

Baseline characteristics were similar, except for higher age and a lower prevalence of dyslipidemia in the AF group. The incidence of a totally occlusive ISR was higher in the AF group than in the sinus group (29.5% vs. 14.6%, *p*=0.004). A multiple Cox regression model suggested that presence of AF (hazard ratio, 2.10) and CTO lesion (hazard ratio, 1.97) which were the independent predictors of a totally occlusive ISR within 3 years. The incidence of ALI was significantly higher in the AF group than in the sinus group (3.9% vs. 0%, *p*=0.0001). In the AF group, the introduction of an anticoagulant did not prevent the occurrence of totally occlusive ISR (*p*=0.71) for ALI (*p*=0.79).

**Conclusions:**

AF is independently associated with totally occlusive ISR of FP stents; however, anticoagulant use does not prevent stent occlusion.

## 1. Introduction

Atrial fibrillation (AF) is an independent risk factor for ischemic stroke, which affected patients being at 5 times the risk of people with sinus rhythm [1, 2]. The prevalence of AF, currently 0.5–1% in the general population, is increasing [3, 4]. Prior studies have reported that the prevalence of peripheral artery disease (PAD) among AF-diagnosed patients is 4–17% [5, 6]. Patients with combined AF and PAD are not rare in clinical practice in this era.

In patients with AF, levels of coagulation system molecular markers including fibrinogen and plasma tissue plasminogen activator were reportedly increased in the peripheral blood regardless of the history of embolic events [7, 8].

AF was also associated with reduced cardiac function and beat-to-beat variations in stroke volume [9]. AF decreased total cerebral blood flow to the brain and disturbed perfusion of the brain tissue compared with the sinus group [10]. Blood flow to the peripheral artery was reduced, similar to cerebral blood flow in patients with AF. Reduced blood flow to the peripheral artery defined as run-off grade was independently associated with the primary patency of stents implanted in femoropopliteal (FP) lesions [11].

We assumed that a prothrombotic state, beat-to-beat variability, and reduced blood flow to the peripheral artery might negatively affect the patency of stents implanted in FP lesions in patients with AF.

## 2. Aim

We aimed to assess the clinical impact of AF on FP stent patency focusing on class III occlusive in-stent restenosis (ISR) susceptible to recurrent ISR [12].

## 3. Methods

### 3.1. Study Population

**Figure 1:**
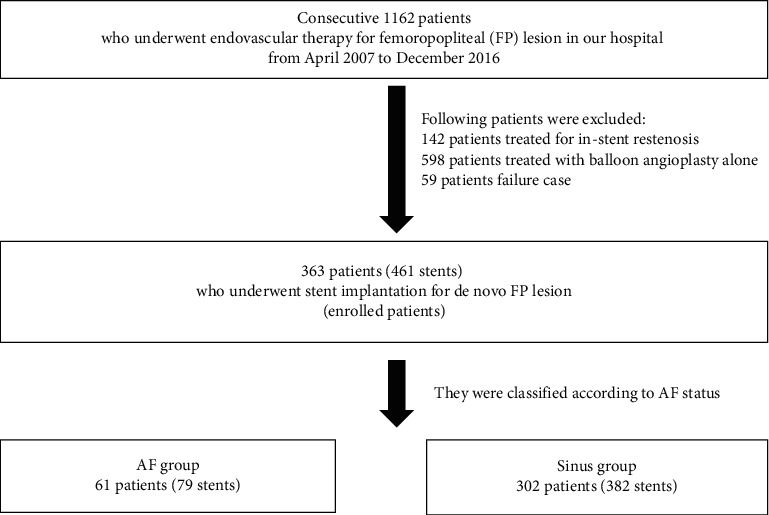
Study design.

Our study was a single center, retrospective, observational study (Saiseikai Yokohama City Eastern Hospital, Yokohama, Kanagawa, Japan). The study design is shown in Figure 1. We performed endovascular therapy (EVT) for FP lesions in 1162 consecutive patients in our hospital between April 2007 and December 2016. After the exclusion of patients who were treated for ISR (142 patients), treated with balloon angioplasty alone including a drug-coated balloon (598 patients) and failure cases (59 patients), 363 patients (461 stents) who underwent stent implantation for de novo lesions were enrolled in this study. The patients were divided into two groups according to AF status: AF group, *n* = 61 (79 stents) and sinus group, *n* = 302 (382 stents).

The study protocol was developed in accordance with the Declaration of Helsinki.

### 3.2. Interventional Procedure

The crossover or ipsilateral approach was used for EVT. Sheaths (6-Fr or 7-Fr) were inserted and unfractionated heparin (5000 U) was injected intra-arterially. The guidewire was passed through the true lumen of the vessel whenever possible using the intraluminal approach. In cases of failure to pass the guidewire using the antegrade approach alone, we switched to a retrograde approach via the popliteal or tibial artery [13, 14].

After a 0.014-inch guidewire was passed through the target lesion, intravascular ultrasound images were recorded to calculate vessel diameter. A stent with a diameter 1–2 mm larger than the reference vessel diameter proximal to the target lesion was selected. Pre- and postdilatations were routinely performed. The implanted stent (bare-metal, drug-eluting, or VIABAHN) was left to the operator's discretion.

### 3.3. Antithrombotic Therapy

Dual-antiplatelet therapy (DAPT) consisting of aspirin (100 mg/day) plus clopidogrel (75 mg/day), ticlopidine (200 mg/day), or cilostazol (200 mg/day) was started at least 3 days before stent implantation and was continued for at least 2 months afterward. Patients with high bleeding risk underwent stent implantation under single-antiplatelet therapy (SAPT) at the discretion of operator.

Anticoagulation for patients with AF was introduced according to the risk of thromboembolic events score, CHADS (2) score [15]. Warfarin or a direct oral anticoagulant (DOAC) was selected according to the renal function and other comorbidities. We did not aggressively introduce warfarin to patients on hemodialysis because it does not decrease stroke risk and negatively affects bleeding risk [16]. Dual antithrombotic therapy (OAC + SAPT) could be used in patients with AF.

### 3.4. Definition

Prior to initiation of the EVT procedure, information regarding selected risk factors was obtained from the patients' hospital records. The following baseline clinical characteristics were collected: age, sex, hypertension, dyslipidemia, diabetes mellitus, past or current smoking status, and prior history of cardiocerebrovascular events.

Cardiac function was evaluated by echocardiography using the modified Simpson method. Low cardiac systolic function was defined as an ejection fraction <40% [17].

Lesion calcification was assessed angiographically using the novel peripheral artery calcification scoring system (PACSS) [18]. Run-off grade 0–3 was assessed by angiography after stent placement [11]. Poor run-off was defined as run-off in zero or one below-the-knee (BTK) vessel [19].

The presence of AF was confirmed by ECGs during hospitalization. A known history of paroxysmal AF was recorded from past medical history or patient reporting. In this study, patterns of AF (paroxysmal, persistent, long-standing persistent, and permanent) were not distinguished for the following reasons: (1) AF type was not associated with thromboembolic events; and (2) precise AF duration is unknown for many cases in real clinical practice [20, 21].

Angiographic patterns were evaluated by Tosaka classification with class III which was defined as totally occlusive ISR [12]. Acute limb ischemia (ALI) was defined as the simultaneous occurrence of acute leg pain and a blood flow disturbance in an FP stent confirmed using any imaging modality [22].

### 3.5. Study Endpoints

The primary endpoint of this study was the incidence of totally occlusive ISR within 3 years after stent implantation. Occlusive ISR was confirmed with duplex ultrasonography, computed tomography, and angiography. The secondary endpoint was the incidence of ALI due to FP stent occlusion.

### 3.6. Statistical Analysis

Continuous variables are expressed as mean ± standard deviation. Categorical variables are expressed as numbers and percentages. Continuous variables were compared using the unpaired student's t-test or the Wilcoxon rank sum test according to their distribution. The chi-square test or Fisher's exact test was used to compare categorical variables. Kaplan-Meier analysis was performed using the log-rank test to compare the incidence of totally occlusive ISR between the AF and sinus groups. A Cox regression analysis was performed to obtain the hazard ratio for totally occlusive ISR. Thereafter, a multivariate analysis was performed using the variables with *p* values <0.10 on univariate analysis to examine their independent associations with total occlusive ISR. The incidence of ALI was evaluated using the chi-square test because the small number of events was insufficient for Kaplan-Meier analysis. The protective efficacy of anticoagulation in AF patients for ALI was evaluated using logistic analysis. All statistical analyses were performed using JMP® 10 (SAS Institute Inc., Cary, NC, USA). *p* values <0.05 were considered statistically significant.

## 4. Results

### 4.1. Patient Characteristics

Patient characteristics are shown in Table 1. Patients in the AF group had a higher mean age (76 ± 9 vs. 73 ± 9, *p*=0.01) and a lower prevalence of dyslipidemia (31% vs. 42%, *p*=0.002). DAPT was introduced in both groups (AF group, 74%; sinus group, 83%; *p*=0.15). Anticoagulants were more frequently used in the AF group (62% vs. 3%, *p* < 0.001). Warfarin and DOAC were used in 28% and 34% of patients in the AF group versus 2% and 1% of patients in the sinus group, respectively. Anticoagulants were prescribed in the sinus group for postvalve replacement (7 patients), protein deficiency (1 patient), and unknown reasons (1 patient). Echocardiography findings were similar between the two groups including the percentage of patients with low cardiac systolic function.

### 4.2. Lesion Characteristics

Lesion characteristics are shown in Table 2. There were no significant differences in lesion characteristics between the AF and sinus groups. Totally, occlusive lesions and Trans-Atlantic Inter-Society Consensus II (TASC) C/D lesions accounted for almost half of the lesions in both groups. Severe calcification defined as PACCS grade 4 was observed in one-tenth of patients in each group. Run-off grade (grade 0/1/2 : 11%/65%/24% in AF group vs. 9%/69%/22% in sinus group, *p*=0.71) and poor BTK run-off (36% vs. 27%, *p*=0.22) were similar between the groups. Bare-metal stent was most frequently used, and the percentage was approximately 80%. Mean total stent length (167 ± 101 mm vs. 160 ± 99 mm, *p*=0.60) and mean stent diameter (6.7 ± 0.7 mm vs. 6.7 ± 0.8 mm, *p*=0.32) were similar between the two groups.

### 4.3. Incidence of Totally Occlusive ISR and ALI

**Figure 2:**
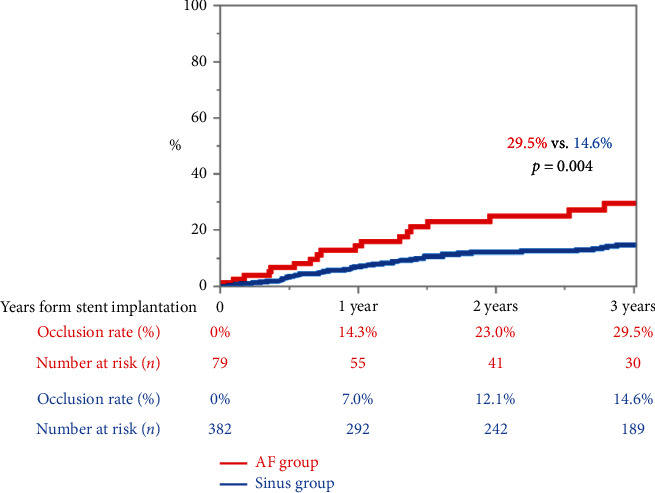
Incidence of totally occlusive in-stent restenosis within 3 years from stent implantation. AF, atrial fibrillation.

Figure 2 shows the incidence of totally occlusive ISR within 3 years after stent implantation. The incidences of totally occlusive ISR at 1, 2, and 3 years in the AF and sinus groups were 14.3% and 7.0%, 23.0% and 12.1%, and 29.5% and 14.6%, respectively; all values were significantly higher in the AF group than in the sinus group (*p*=0.004).

The Cox proportional hazard analysis of predictors for totally occlusive ISR within 3 years is shown in Table 3. On univariate analysis, nonambulatory status, female sex, presence of AF, presence of chronic total occlusion (CTO) lesions, and presence of TASC C/D lesions were associated with a totally occlusive ISR. The multivariate Cox regression model suggested that the presence of AF (hazard ratio, 2.10; 95% CI, 1.18–3.57; *p*=0.01) and the presence of a CTO lesion (hazard ratio, 1.97; 95% CI, 1.02–3.96; *p*=0.04) were independent predictors of a totally occlusive ISR within 3 years.

**Figure 3:**
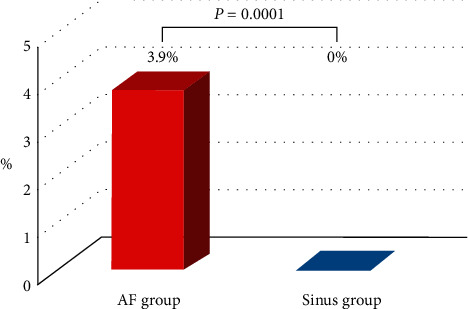
Incidence of acute limb ischemia due to stent occlusion. AF, atrial fibrillation.

The incidence of ALI was significantly higher in the AF group than in the sinus group (3.9% vs. 0%, *p*=0.0001) (Figure 3).

Anticoagulant use showed no protective efficacy against a totally occlusive ISR in patients with AF (relative risk, 0.84; 95% CI, 0.33–2.27; *p*=0.71) or ALI (relative risk (RR), 0.94; 95% CI, 0.59–1.52; *p*=0.79) (Table 4). Even in patients with sinus rhythm, anticoagulation use did not prevent a totally occlusive ISR (RR, 1.01; 95% CI, 0.57–2.03; *p*=0.98).

## 5. Discussion

AF is a serious public health problem because of its increasing incidence and prevalence in the aging population [3]. The increasing clinical impact of AF on stents implanted in the peripheral arteries of patients with comorbid PAD and AF has not been thoroughly discussed, especially in terms of patency.

The present study demonstrated the following: (1) the incidence of totally occlusive ISR of FP stents was significantly higher in the AF group than in the sinus group; (2) the presence of AF was an independent predictor of totally occlusive ISR; (3) the incidence of ALI due to stent occlusion was higher in the AF group than in the sinus group; and (4) anticoagulant use did not show a preventive effect against totally occlusive ISR or ALI.

Predictors of a totally occlusive ISR were reported as female sex, CLI, and TASC C/D, but the presence of AF was not considered in this study [23].

An adverse effect of AF on the peripheral artery is a prothrombotic state induced by the arrhythmia itself [7, 8]. Partial development of organic stenosis in a stent followed by thrombus formation may be a potential mechanism of the development of totally occlusive ISR [24]. Considering this mechanism, anticoagulation should prevent totally occlusive ISR by interfering with primary thrombus formation in AF patients, although anticoagulation was not a protective factor against totally occlusive ISR in our study. In-stent thrombosis is the result of a more complex process involving cellular membrane receptors, platelets, and coagulation cascade. OAC only block coagulation cascade while not limiting platelet adhesion and aggregation which are the main determinants of in-stent thrombosis. Anticoagulation use might be not enough to prevent the formation of in-stent thrombosis.

Another study also reported that thrombus formation accelerated in the atrium but no significant changes in the peripheral blood (saphenous vein) in a dog model of AF [25]. It should be clarified more clearly whether the thrombotic state was induced by AF in the peripheral artery, especially inside implanted stents.

The other adverse effect of AF on the peripheral artery was hypoperfusion induced by decreased cardiac output and decreased blood flow [26]. Patients with persistent AF also had decreased perfusion of the brain tissue than those with sinus rhythm [10]. The mechanism of flow disturbance induced by AF was not clearly elucidated but considered related with beat-to-beat variations in stroke volume in AF. A highly restricted blood flow to the tissue also induces microvascular damage. The microvascular damage is a likely cause of a poor blood flow [27], as it is a peripheral circulatory disorder. A restricted blood flow and reduced microvascular perfusion are negatively associated with each other in the peripheral arteries of patients with AF.

Periphral blood flow evaluated with run-off grade and BTK run-off vessels was similar between two groups. The relationship between BTK artery and stent patency was still controversial [28, 29]. Moreover, microvascular function had not been clearly demonstrated as an independent predictor of ISR and occlusion in both coronary and peripheral diseases. Tissue perfusion in patients with PAD could be evaluated using modalities such as contrast-enhanced ultrasonography, magnetic resonance imaging perfusion imaging, and near-infrared spectroscopy [30–32]. The relationship between AF and lower-limb perfusion only evaluated with imaging modalities and could be a solution for the frequent occurrence of totally occlusive ISR in AF patients.

In individuals undergoing elective cardioversion for AF, the restoration and maintenance of sinus rhythm were associated with improved brain perfusion and cerebral blood flow [33]. The effect of maintaining sinus rhythm on patency was not reported previously and obscure but improved tissue perfusion and blood flow could positively prevent stent occlusion. Flow grade reportedly influenced heart rate (HR), and an increasing HR significantly decreased the thrombosis in myocardial infarction frame count [34]. Optimal HR control for patients with AF could help maintain peripheral artery flow and stent patency.

In this study, the incidence of ALI due to FP stent occlusion was higher in the AF group. A previous study reported that FP stent thrombosis was associated with the treatment of CTO and ISR lesions, but AF status was not evaluated [35]. Anticoagulation use was not protective against ALI in this study, so that clinical impact and mechanism of AF on ALI remain unclear.

The limitations of this study were as follows: first, only a relatively small number of patients in AF group (*n* = 61) were enrolled compared with patients in the sinus group (*n* = 302). Clinical impact of AF on stent patency should be evaluated in large sample cohort. Second, anticoagulation has been administrated in only 62% of AF patients. This under administration was caused by patients' background such as the elder, bleeding risk, and hemodialysis but could obscure the relationship between AF and stent patency. A prospective study with strict exclusion criteria should be planned for solutions.

## 6. Conclusions

AF is independently associated with totally occlusive ISR of FP stents. Anticoagulation use does not prevent stent occlusion, thus confirming that coagulation cascade is not the unique determinant of in-stent thrombus formation.

## Figures and Tables

**Table 1:** Patient's characteristics.

Variables	AF group (*n* = 61)	Sinus group (*n* = 302)	*P* value
Age	76 ± 9	73 ± 9	0.01
Female, *n* (%)	21 (34)	100 (33)	0.84
Ambulatory	49 (80)	256 (85)	0.39
Hypertension, *n* (%)	50 (82)	233 (77)	0.41
Dyslipidemia, *n* (%)	19 (31)	128 (42)	0.002
Diabetes mellitus, *n* (%)	26 (43)	166 (55)	0.08
Smoking, *n* (%)	6 (10)	42 (15)	0.49
Critical limb ischemia, *n* (%)	18 (30)	89 (31)	0.83
Prior PCI, *n* (%)	23 (37)	101 (33)	0.52
Prior CABG, *n* (%)	2 (3)	6 (2)	0.53
Prior MI, *n* (%)	3 (5)	10 (3)	0.54
Prior CVD, *n* (%)	2 (3)	12 (4)	0.8
CKD on HD, *n* (%)	8 (13)	50 (17)	0.72
Medication			
Dual-antiplatelet therapy, *n* (%)	31 (51)	226 (75)	<0.001
Aspirin, *n* (%)	39 (64)	250 (83)	—
Clopidgrel, *n* (%)	28 (46)	152 (51)	—
Prasuglel, *n* (%)	4 (7)	2 (1)	—
Ticlopidine, *n* (%)	6 (10)	33 (11)	—
Cilostazol, *n* (%)	13 (21)	109 (36)	—
Anticoagulation, *n* (%)	38 (62)	10 (3)	<0.001
Echographic findings			
Dd (mm)	45 ± 9	45 ± 8	0.81
Ds (mm)	32 ± 10	31 ± 8	0.31
EDV (ml)	83 ± 36	86 ± 40	0.68
ESV (ml)	47 ± 31	39 ± 28	0.41
Stroke volume (ml)	43 ± 14	47 ± 29	0.14
Ejection fraction (%)	55 ± 13	58 ± 11	0.16
Low EF (<40%)	11 (18%)	32 (11)	0.11

AF, atrial fibrillation; PCI, percutaneous coronary intervention; CABG, coronary artery bypass graft; Mi, myocardial infarction; CVD, cerebrovascular disease; CKD, chronic kidney disease; HD, hemodialysis; DOAC, direct oral anticoagulants; EDV, end-diastolic volume; ESV, end-systolic volume; EF, ejection fraction.

**Table 2:** Lesion's characteristics.

Variables	AF group (*n* = 79)	Sinus group (*n* = 382)	*P* value
Chronic total occlusion, *n* (%)	41 (52)	197 (52)	0.96
TASC C/D lesions, *n* (%)	39 (49)	179 (47)	0.68
Calcification, *n* (%)			
PACCS grade 0-1, *n* (%)	20 (25)	71 (19)	0.39
PACCS grade 2, *n* (%)	36 (46)	213 (56)	—
PACCS grade 3, *n* (%)	14 (18)	61 (16)	—
PACCS grade 4, *n* (%)	9 (11)	36 (9)	—
Run-off grade, *n* (%)			
Grade 0, *n* (%)	9 (11)	34 (9)	—
Grade 1, *n* (%)	51 (65)	264 (69)	0.71
Grade 2, *n* (%)	19 (24)	84 (22)	—
Poor run-off, *n* (%)	17 (36)	66 (27)	0.22
Kind of stents, *n* (%)			
Bare-metal stent, *n* (%)	66 (84)	319 (83)	0.91
Drug-eluting stent, *n* (%)	13 (16)	62 (16)	—
VIABAHN, *n* (%)	0 (0)	1 (1)	—
Total stent length (mm)	167 ± 101	160 ± 99	0.60
Stent diameter (mm)	6.7 ± 0.7	6.7 ± 0.8	0.64
Stenting on P1 segment, *n*(%)	19 (24)	73 (19)	0.32

AF, atrial fibrillation; TASC, Trans-Atlantic Inter-Society Consensus; PACCS, peripheral artery calcification scoring system.

**Table 3:** Predictors of totally occlusive ISR within 3 years.

Variables	Univariate analysis			Multivariate analysis		
	Hazard ratio	95% CI	*P* value	Hazard ratio	95% CI	*P* value
Age (per 1 increase)	1.01	0.97–1.06	0.12	—	—	—
Female	1.78	1.08–2.92	0.02	1.63	0.99–2.69	0.05
Nonambulatory	2.27	1.20–4.01	0.01	1.68	0.88–2.99	0.11
Dyslipidemia	1.41	0.75–1.74	0.26	—	—	—
Diabetes mellitus	1.12	0.68–1.86	0.65	—	—	—
Hemodialysis	0.76	0.32–1.55	0.48	—	—	—
Atrial fibrillation	2.31	1.24–3.75	0.008	**2.1**	**1.18–3.57**	**0.01**
Low EF (<40%)	1.58	0.50–4.82	0.23	—	—	—
Critical limb ischemia	1.54	0.89–2.57	0.12	—	—	—
Dual-antiplatelet therapy	1.36	0.78–2.52	0.28	—	—	—
Chronic total occlusion	3.01	1.75–5.48	<0.001	**1.97**	**1.02–3.96**	**0.04**
TASC C/D lesions	2.91	1.72–5.09	<0.001	1.81	0.97–3.51	0.06
PACCS grade 4	0.79	0.21–1.68	0.45	—	—	—
Run-off grade 0	1.42	0.71–2.53	0.41	—	—	—
Poor run-off	1.26	0.63–2.40	0.26	—	—	—
Drug-eluting stent implantation	1.03	0.55–2.15	0.93	—	—	—

EF, ejection fraction; TASC, Trans-Atlantic Inter-Society Consensus; PACCS, peripheral artery calcification scoring system.

**Table 4:** Anticoagulant use did not prevent totally occlusive ISR and acute limb ischemia in patients with AF.

Anticoagulant use	Risk ratio	95% CI	*P* value
For totally occlusive ISR	0.84	0.33–2.27	0.71
For acute limb ischemia	0.94	0.59–1.52	0.79

ISR, in-stent restenosis; AF, atrial fibrillation.

## Data Availability

The data used to support the findings of the study can be made available upon request to the corresponding author.

## References

[1] Wolf P. A., Dawber T. R., Thomas H. E., Kannel W. B. (1978). Epidemiologic assessment of chronic atrial fibrillation and risk of stroke: the fiamingham study. *Neurology*.

[2] Tanaka H., Hayashi M., Date C. (1985). Epidemiologic studies of stroke in Shibata, a Japanese provincial city: preliminary report on risk factors for cerebral infarction. *Stroke*.

[3] Go A. S., Hylek E. M., Phillips K. A. (2001). Prevalence of diagnosed atrial fibrillation in adults. *JAMA*.

[4] Murphy N. F., Simpson C. R., Jhund P. S. (2007). A national survey of the prevalence, incidence, primary care burden and treatment of atrial fibrillation in Scotland. *Heart*.

[5] Violi F., Lip G. Y. H., Basili S. (2012). Peripheral artery disease and atrial fibrillation: a potentially dangerous combination. *Internal and Emergency Medicine*.

[6] Violi F., Daví G., Hiatt W. (2013). Prevalence of peripheral artery disease by abnormal ankle-brachial index in atrial fibrillation. *Journal of the American College of Cardiology*.

[7] Kahn S. R., Solymoss S., Flegel K. M. (1997). Nonvalvular atrial ﬁbrillation: evidence for a prothrombotic state. *Canadian Medical Association Journal*.

[8] Kahn S. R., Solymoss S., Flegel K. M. (1997). Increased tissueplasminogen activator levels in patients with nonvalvular atrial ﬁbrillation. *Canadian Medical Association Journal*.

[9] Wyse D. G. (2008). Therapeutic considerations in applying rate control therapy for atrial fibrillation. *Journal of Cardiovascular Pharmacology*.

[10] Gardarsdottir M., Sigurdsson S., Aspelund T. (2018). Atrial fibrillation is associated with decreased total cerebral blood flow and brain perfusion. *EP Europace*.

[11] Hiramori S., Soga Y., Tomoi Y., Tosaka A. (2014). Impact of runoff grade after endovascular therapy for femoropopliteal lesions. *Journal of Vascular Surgery*.

[12] Tosaka A., Soga Y., Iida O. (2012). Classification and clinical impact of restenosis after femoropopliteal stenting. *Journal of the American College of Cardiology*.

[13] Baril D. T., Chaer R. A., Rhee R. Y., Makaroun M. S., Marone L. K. (2010). Endovascular interventions for TASC II D femoropopliteal lesions. *Journal of Vascular Surgery*.

[14] Tokuda T., Hirano K., Muramatsu T., Tsukahara R., Nakano M. (2014). A sheathless retrograde approach via the popliteal artery is useful and safe for treating chronic total occlusions in the superficial femoral artery. *Journal of Endovascular Therapy*.

[15] Gage B. F., Waterman A. D., Shannon W., Boechler M., Rich M. W., Radford M. J. (2001). Validation of clinical classification schemes for predicting stroke. *JAMA*.

[16] Shah M., Avgil Tsadok M., Jackevicius C. A. (2014). Warfarin use and the risk for stroke and bleeding in patients with atrial fibrillation undergoing dialysis. *Circulation*.

[17] Yancy C. W., Jessup M., Bozkurt B. (2013). 2013 ACCF/AHA guideline for the management of heart failure: a report of the American college of cardiology foundation/American heart association task force on practice guidelines. *Circulation*.

[18] Okuno S., Iida O., Shiraki T. (2016). Impact of calcification on clinical outcomes after endovascular therapy for superficial femoral artery disease. *Journal of Endovascular Therapy*.

[19] Soga Y., Iida O., Hirano K. (2011). Utility of new classification based on clinical and lesional factors after self-expandable nitinol stenting in the superficial femoral artery. *Journal of Vascular Surgery*.

[20] Camm A. J., Lip G. Y., De Caterina R. (2012). 2012 focused update of the ESC Guidelines for the management of atrial fibrillation: an update of the 2010 ESC Guidelines for the management of atrial fibrillation. Developed with the special contribution of the European Heart Rhythm Association. *European Heart Journal*.

[21] Hohnloser S. H., Pajitnev D., Pogue J. (2007). Incidence of stroke in paroxysmal versus sustained atrial fibrillation in patients taking oral anticoagulation or combined antiplatelet therapy. *Journal of the American College of Cardiology*.

[22] Creager M. A., Kaufman J. A., Conte M. S. (2012). Acute limb ischemia. *New England Journal of Medicine*.

[23] Dohi T., Iida O., Soga Y. (2014). Incidence, predictors, and prognosis of in-stent occlusion after endovascular treatment with nitinol stents for femoropopliteal lesions. *Journal of Vascular Surgery*.

[24] Ishihara T., Iida O., Okamoto S. (2017). Potential mechanisms of in-stent occlusion in the femoropopliteal artery: an angioscopic assessment. *Cardiovascular Intervention and Therapeutics*.

[25] Yamada S., Hirao D., Miura N. (2018). Comparison of chronological changes in blood characteristics in the atrium and peripheral vessels after the development of non-valvular atrial fibrillation. *Thrombosis Research*.

[26] Diener H.-C., Hart R. G., Koudstaal P. J., Lane D. A., Lip G. Y. H. (2019). Atrial fibrillation and cognitive function. *Journal of the American College of Cardiology*.

[27] Akasaka T., Yoshida K., Kawamoto T. (2000). Relation of phasic coronary flow velocity characteristics with TIMI perfusion grade and myocardial recovery after primary percutaneous transluminal coronary angioplasty and rescue stenting. *Circulation*.

[28] Dearing D. D., Patel K. R., Compoginis J. M., Kamel M. A., Weaver F. A., Katz S. G. (2009). Primary stenting of the superficial femoral and popliteal artery. *Journal of Vascular Surgery*.

[29] Lee J. J., Katz S. G. (2012). The number of patent tibial vessels does not influence primary patency after nitinol stenting of the femoral and popliteal arteries. *Journal of Vascular Surgery*.

[30] Duerschmied D., Zhou Q., Rink E. (2009). Simplified contrast ultrasound accurately reveals muscle perfusion deficits and reflects collateralization in PAD. *Atherosclerosis*.

[31] Suo S., Zhang L., Tang H. (2018). Evaluation of skeletal muscle microvascular perfusion of lower extremities by cardiovascular magnetic resonance arterial spin labeling, blood oxygenation level-dependent, and intravoxel incoherent motion techniques. *Journal of Cardiovascular Magnetic Resonance*.

[32] Boezeman R. P., Boersma D., Wille J. (2016). The significance of regional hemoglobin oxygen saturation values and limb-to-arm ratios of near-infrared spectroscopy to detect critical limb ischemia. *Vascular*.

[33] Gardarsdottir M., Sigurdsson S., Aspelund T. (2020). Improved brain perfusion after electrical cardioversion of atrial fibrillation. *EP Europace*.

[34] Abaci A., Oguzhan A., Eryol N. K., Ergin A. (1999). Effect of potential confounding factors on the thrombolysis in myocardial infarction (TIMI) trial frame count and its reproducibility. *Circulation*.

[35] Banerjee S., Sarode K., Mohammad A. (2016). Femoropopliteal artery stent thrombosis: report from the excellence in peripheral artery disease registry. *Circulation: Cardiovascular Interventions*.

